# Supplementary material for: Sustained Endurance Training Leads to Metabolomic Adaptation
Source: Metabolites. 2022 Jul 16;12(7):658. doi: 10.3390/metabo12070658 (PMC9323347; doi:10.3390/metabo12070658)
Supplement: Supplementary file 1 [file metabolites-12-00658-s001.zip › metabolites-1803773-supplementary.pdf]

Figure S1

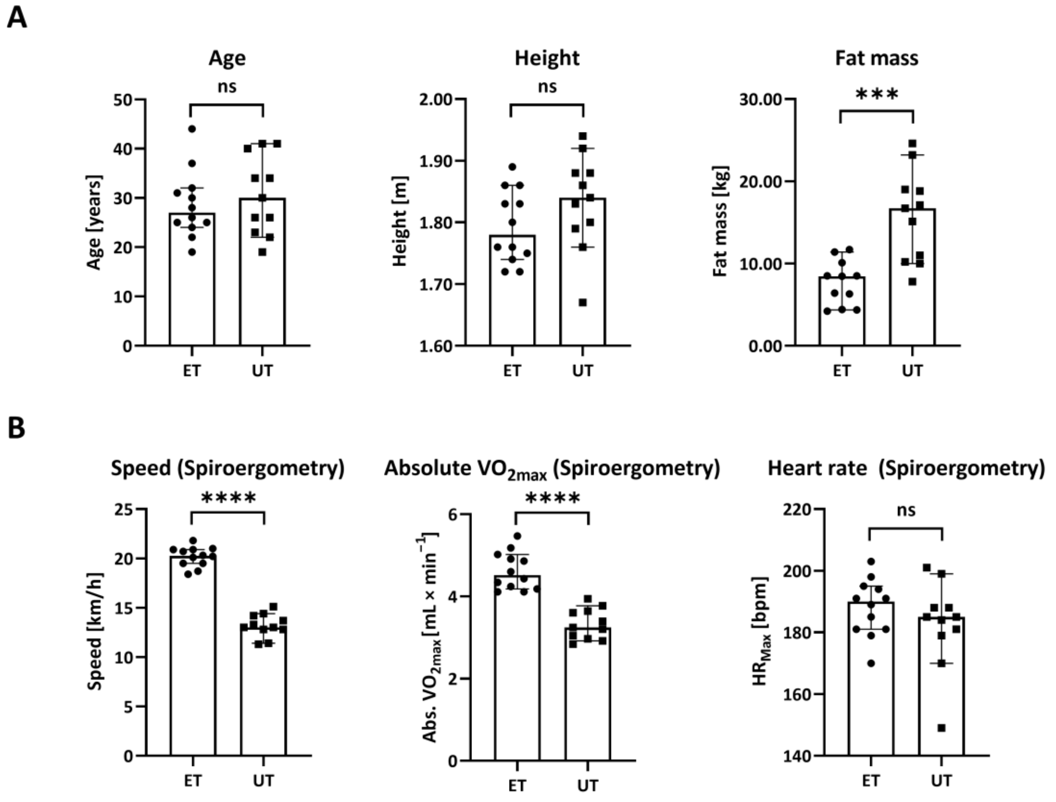

Figure S1. Additional participants´ details. (**A**) Physiological parameters, i.e. age, height and fat mass, for all volunteers that were grouped into ET (n = 12) or UT (n = 11). (**B**) Data further describing the speed, absolute VO_{2max} as well as the heart rate during spiroergometry for all participants. Data have been statistically analyzed by an unpaired, non-parametric, two-tailed t-test (Mann–Whitney test). ***: $p \leq 0.001$; ****: $p \leq 0.0001$.
